# Analysis of Adverse Events Post-13-Valent Pneumococcal Vaccination among Children in Hangzhou, China

**DOI:** 10.3390/vaccines12060576

**Published:** 2024-05-25

**Authors:** Jing Wang, Jian Du, Yan Liu, Xinren Che, Yuyang Xu, Jiayin Han

**Affiliations:** Department of Expanded Program on Immunization, Hangzhou Center for Disease Control and Prevention, Hangzhou 310021, China; jackiewong203@163.com (J.W.); dujianhzcdc@163.com (J.D.); 15888831749@163.com (X.C.); jlt422519@163.com (Y.X.); hanjiayin0128hz@163.com (J.H.)

**Keywords:** adverse events following immunization, 13-valent pneumonia vaccine, vaccine safety

## Abstract

With the widespread use of the 13-valent pneumonia vaccine (PCV13) in China, monitoring adverse events following immunization (AEFIs) is critical. We conducted a descriptive analysis of the AEFI occurrences reported within Hangzhou between the years 2020 and 2023, including the temporal trend of case reports and variables such as sex, age, type of PCV13, dose number, type of reporter, cause-specific classification, severity, and onset from vaccination. Vaccine safety signals were analyzed using reporting odds ratios (RORs). Over the 4 years analyzed in the study, 2564 AEFI cases were reported, including seven severe cases. Most AEFIs occurred within 0–1 days after vaccination (2398, 93.53%), with over half affecting infants aged 1.5–6 months of age. No statistically significant difference was observed between PCV13-TT and PCV-CRM197. Seasonal differences in AEFI reports were noted. Positive signals were detected for fever (ROR-1.96SE: 1.64) and persistent crying (ROR-1.96SE: 1.61). Four serious AEFI cases were coincidental events, while three others were considered vaccine-related cases (including one case each of allergic reaction, febrile seizure, and thrombocytopenia). The safety and tolerability of PCV13 are good, and attention should be paid to severe AEFIs, as well as long-term safety disparities between different types of PCV13.

## 1. Introduction

*Streptococcus pneumoniae*, a widespread pathogenic bacterium, is the main cause of various severe health conditions, including bacterial pneumonia, otitis media, and meningitis [1,2]. These diseases pose a significant threat to the health of infants and older adults, affecting their quality of life and potentially endangering their lives [3]. Because of the widespread influence and serious harm caused by this pathogenic bacterium, the development and application of *Streptococcus pneumoniae* vaccines have gained significant global importance, making it a prominent research topic in the field of public health.

The 13-valent pneumonia vaccine (PCV13) has attracted considerable attention because of its wide serotype coverage and high efficiency. This vaccine targets 13 serotypes of *Streptococcus pneumoniae*, including 1, 3, 4, 5, 6A, 6B, 7F, 9V, 14, 18C, 19A, 19F, and 23F, preventing disease by inducing the body to produce specific antibodies [4]. PCV13 is a polysaccharide protein-binding vaccine with the widest serotype coverage and is effective in building immunity in early childhood, especially for those below 2 years of age. The 13-valent pneumococcal vaccine is utilized in developed countries such as the United States and South Korea, as well as in less developed countries, such as Tanzania and Nepal, to mitigate the impact of invasive pneumococcal disease (IPD) [1,5,6,7]. A cross-sectional study from Tanzania showed a 50% reduction in the incidence of pneumococci belonging to PCV13 types in children after vaccination [7]. Additionally, administering the PCV13 leads to economically advantageous outcomes compared to not vaccinating, especially among children under 5 years of age [8]. Research conducted in mainland China revealed that the supplementary expense associated with achieving each quality-adjusted life-year by administering a dose of the novel PCV13 to children aged between 2 and 5 years was approximately USD 2417 when juxtaposed with alternative immunization regimens [9].

Although the effectiveness and safety of PCVs in normal and high-risk populations with low immunity have been widely recognized [10], severe adverse events have been reported in some vaccinated individuals, such as severe allergic reactions and febrile seizures. These adverse reactions may be related to vaccine composition, individual physiological differences, and other factors, as demonstrated in several studies [5,11,12,13]. PCV13 was approved for marketing in China in 2017. Hu et al. conducted a vaccination safety study in infants soon in Zhejiang after the PCV13 was approved [14]. However, considering the Weber effect, the initial reporting rate of adverse events after the launch of a new vaccine may be relatively high but will gradually stabilize over time [14]. Therefore, after the launch of the PCV13 and once the sensitivity of adverse event reporting reaches a stable state, research should be conducted to reflect the actual occurrence of adverse reactions to the vaccine more accurately and to better evaluate the safety of the vaccine in the real world. This is vital for evaluating vaccine safety and improving public confidence in vaccines.

Hangzhou, located in the southeastern coastal region of China, is one of the most economically developed cities in the country, with a population of approximately 10 million permanent residents. In 2017, the PCV13 was introduced as one of 29 non-immunization program vaccines in the city, priced at approximately USD 80–100 per dose for children aged 6 weeks to 5 years [15]. Owing to its high cost and the characteristics of the inoculated population, the increasing PCV13 vaccination rate has attracted widespread public attention.

Acknowledging the insufficiency of existing research on adverse events following the PCV13 in Chinese children, we aimed to analyze the occurrence and reporting rate of adverse events following PCV13 vaccination in Hangzhou, China, from 2020 to 2023 and compare these data with the reporting rate of adverse events following immunization (AEFIs) after other vaccinations during the same period. This study intended to assess the safety of the PCV13 and explore its potential adverse effects in China.

## 2. Materials and Methods

### 2.1. AEFI Surveillance System

The surveillance system for AEFIs in China began in 2005, and in 2008, it underwent a significant transformation to become a comprehensive nationwide online platform known as the Chinese National AEFI Information System (CNAEFIS) [16]. CNAEFIS operates according to China’s national AEFI guidelines, which are supported by various laws, including the Law of the People’s Republic of China on the Prevention and Treatment of Infectious Diseases, Drug Administration Law of the People’s Republic of China, Vaccine Administration Law of the People’s Republic of China, National Immunization Program Operational Guidelines, Regulations on the Prevention and Response to Public Health Emergencies, and other laws and regulations [16]. The objectives of CNAEFIS include monitoring abnormal changes in known AEFIs, identifying potential risk factors, evaluating the safety of newly marketed vaccines, and providing evidence to improve vaccine quality and vaccination services [14].

### 2.2. Data Extraction

Two types of PCV13 were used for voluntary selection in Hangzhou: PCV13-TT and PCV13-CRM197. PCV13-TT uses the tetanus toxoid carrier protein and is produced in China. In contrast, PCV13-CRM197 uses the CRM197 carrier protein and is produced by a company based in Ireland. Both vaccinations follow a four-dose series: a basic immunization dose at 2, 4, and 6 months of age, followed by a booster dose at 12 to 15 months of age. The primary immunization cycle can begin as early as 6 weeks of age, with a minimum inter-dose gap of 4 weeks throughout the sequence. Although these two types of vaccines have the same immunization protocol and principles, it is generally not recommended that they be used interchangeably in real-world vaccinations.

The study extracted all AEFI cases reported after vaccination with PCV13 reported from 1 January 2020 to 31 December 2023, from CNAEFIS according to the onset time. These cases were evaluated and classified by an AEFI expert group. The reported cases in CNAEFIS included sex, age, type of PCV13, dose number, cause-specific classification, and severity, and originated from vaccination clinics or the CDCs. Age grouping refers to the grouping of two types of vaccines in clinical trials and post-marketing safety monitoring in China. The time interval from vaccination to illness onset was grouped according to the Chinese National AEFI guidelines. Vaccination data were sourced from an internet-based personal vaccination system in Hangzhou during the same period.

### 2.3. Definitions of AEFI

According to the WHO, an AEFI is defined as any untoward medical occurrence following immunization and does not necessarily have a causal relationship with vaccine use [17]. AEFIs can be divided into (a) vaccine product-related reactions, (b) vaccine quality defect-related reactions, (c) immunization error-related reactions, (d) immunization anxiety-related reactions, and (e) coincidental events [17]. AEFIs can be systematically classified based on their severity. Specifically, AEFIs were categorized into two distinct groups: non-severe and severe. Non-severe AEFIs included those that either required no intervention or may have necessitated medical consultations and led to minor disruption of daily activities or loss of work time. In contrast, serious AEFIs were described as adverse health episodes that had significant clinical repercussions, such as fatality occurrences, patient hospitalization, extended stays in healthcare settings, sustained or major disabilities or functional limitations, life-threatening conditions, or the appearance of congenital disorders [14,16]. In China, we assembled groups of AEFI experts to evaluate the causal relationships of cases and classify them according to the WHO manual. These experts were from diverse fields, including clinical medicine, epidemiology, pharmacy, pathology, adverse drug reaction offices, and other related fields.

### 2.4. Data Analysis

The database was compiled and organized using Microsoft Excel (Microsoft Corporation, Redmond, WA, USA). The temporal trend of AEFIs following the PCV13 was presented by combining monthly AEFI case report numbers with reporting rates. The reporting rates were categorized by quarter and analyzed using seasonal decomposition in the forecasting module of SPSS (Version 22.0, IBM Corporation, Armonk, NY, USA). Descriptive data included variables such as sex, age, type of PCV13, dose, type of reporter, cause-specific classification, severity, and onset of vaccination. The AEFI reporting rate for each variable was calculated by dividing the number of AEFIs during the study period by the number of vaccinations [14]. Quantitative data are described as numbers or rates, and differences were compared using the chi-squared test. Statistical significance was set at *p* < 0.05.

The research methodology for vaccine safety signals involves disproportionality analyses [18,19]. Nevertheless, if the calculated result between a specific vaccine and a particular diagnosis exceeds the expected threshold, it indicates a proportional imbalance, suggesting the emergence of a potential safety signal. This study employs the reporting odds ratio (ROR) for the analysis. If the lower limit of the 95% confidence interval of the ROR is >1, it suggests the presence of a potential safety signal [18].

Multiple diagnoses may coexist in one AEFI case; therefore, the primary diagnosis was used in this study.

## 3. Results

### 3.1. Descriptive Results and Chi-Square

Among the reported cases of AEFIs, 55.81% were male, with an AEFI reporting rate of 29.11 per 10,000 doses, while the female reporting rate was 24.85 per 10,000 doses. More than half of the AEFI cases related to the PCV13 conjugate vaccine occurred in infants aged 1.5 to 6 months, with a reporting rate of 27.83 per 10,000 doses, which then increased to 29.19 per 10,000 doses in children aged 7–12 months and decreased to 24.16 per 10,000 doses in children aged 13–24 months. The highest reporting rate was observed in children older than 2 years (24 months), reaching 29.97 per 10,000 doses, with statistically significant differences in AEFI reporting rates among the various age groups.

Among the two varieties of the PCV13 used in Hangzhou, 1629 cases of AEFIs related to the PCV13-CRM197 were reported, with a reporting rate of 27.13 per 10,000 doses, while 935 cases related to the PCV13-TT were reported, with a similar reporting rate of 26.93 per 10,000 doses, showing no statistically significant difference between the two types. Most of the reported cases originated from vaccination clinics (2499, 97.46%). In terms of inoculation sites, a total of 562 cases of abnormal reactions were reported in children who were vaccinated in the upper arm (22.73 per 10,000 doses), while 2002 cases were reported in children who received thigh muscle inoculations (28.59 per 10,000 doses), showing a statistically significant difference (chi-squared = 23.21, *p* < 0.05). In terms of vaccine doses, the AEFI report rate was highest for the first dose, at 34.11 per 10,000 doses. As the number of doses increased, both the AEFI report numbers and rates decreased gradually to 684 cases (28.59 per 10,000 doses) and 474 cases (20.57 per 10,000 doses), respectively. However, by the fourth dose, the report rate had increased again to reach 23.83 per 10,000 doses. The dose numbers showed a statistically significant difference (chi-squared = 95.12, *p* < 0.05).

According to an expert group’s classification of these reported AEFIs related to the PCV13, 2547 cases were classified as vaccine product-related reactions (3 severe cases and 2544 minor cases), and only 17 cases were classified as coincidental events. Of the 2564 cases of AEFIs, a total of 7 severe cases were reported. Most AEFIs occurred within 0–1 days after vaccination (2398, 93.53%), with the number of cases gradually decreasing as the interval increased (Table 1).

**Figure 1:**
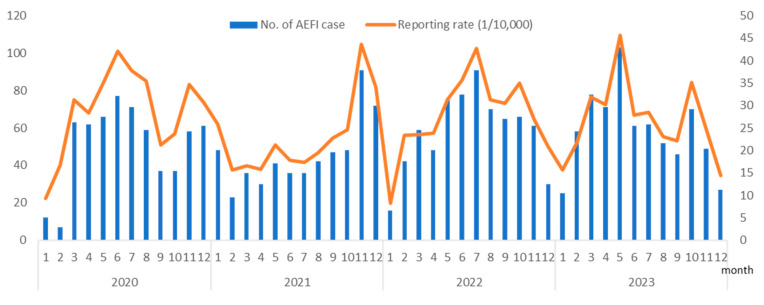
Number of adverse events following immunization (AEFI) cases and reporting rate (1 per 10,000) associated with the 13-valent pneumococcal conjugate vaccine (PCV13) from 2020 to 2023 in Hangzhou, China.

From 1 January 2020 to 31 December 2023, 947,555 doses of PCV13 were administered in Hangzhou, averaging 19,740.73 doses per month. During this study, 2564 AEFI cases related to the PCV13 were reported, resulting in an AEFI reporting rate of 27.06 per 10,000 doses. Serious AEFIs were reported in 2021 (two cases), 2022 (three cases), and 2023 (two cases), with a total reporting rate of 0.07 per 10,000 doses. The highest AEFI reporting rate following the administration of the vaccine was observed in May 2023 at a rate of 45.61 per 10,000 doses, and the lowest was recorded in January 2020 at a rate of 9.26 per 10,000 doses. The year 2020 had the highest AEFI reporting rate at 30.31 per 10,000 doses, followed by 2022 (28.08 per 10,000 doses), 2023 (27.15 per 10,000 doses), and 2021 (23.13 per 10,000 doses). The AEFI reporting rates varied significantly over the 4 years (chi-squared = 22.21, *p* < 0.01). The differences in reporting rates between years are thought to be associated with fluctuations in the level of understanding and attention that vaccine recipients’ guardians have towards vaccination. Seasonal analysis revealed seasonal variations in the PCV13 AEFI reporting rates, with the lowest seasonal factor observed in the first quarter at 82%, whereas in the other three quarters, it was 107.4%, 103.1%, and 107.6%, respectively (Figure 1).

### 3.2. Clinical Diagnosis and ROR

Table 2 shows the clinical diagnosis of 2564 AEFI cases following the administration of the PCV13, with the majority being minor vaccine-related reactions. The most commonly reported AEFI diagnosis was fever, with a reporting rate of 13.04 per 10,000 doses, followed by injection site reactions (1207 cases, 12.74 per 10,000 doses). There were 58 reported cases of rash/urticaria (0.61/10,000), 25 reported cases of gastrointestinal reactions including vomiting and diarrhea (0.26/10,000), and 17 reported cases of persistent crying (0.18/10,000), which are also minor vaccine-related adverse events. The common non-serious coincidental events mainly consisted of fever (eight cases) and cough (three cases).

Among the vaccine product-related severe AEFIs reported, there was one case each of allergic reaction, febrile seizure, and thrombocytopenia. The clinical diagnoses of the four severe coincidental events were cardiovascular, febrile seizures, nervous system reactions, and Kawasaki disease. Positive signals were only observed for fever (ROR-1.96SE: 1.64) and persistent crying (ROR-1.96SE: 1.61).

### 3.3. Serious AEFI Cases

Between 2021 and 2023, seven severe cases were reported among children who were inoculated with the PCV13, with five cases involving males and two involving females. No severe cases were reported in 2020. The majority of the severe cases occurred in children aged < 6 months (five cases), while the oldest case was vaccinated after reaching > 36 months of age. Among the reported cases, three individuals received the PCV13-TT, while the remaining four received the PCV13-CRM197. Severe reactions were reported for all four doses, with the highest number of reports associated with the second dose (four cases), followed by the first dose (two cases), third dose (one case), and fourth dose (one case). In terms of the injection site, three individuals were injected in their arms and four in their thighs.

Most severe reactions developed rapidly; for instance, a nervous system reaction (epilepsy) was reported within one hour post-vaccination. Conversely, thrombocytopenia was not reported until day 15 following vaccination. Following a review by expert panels, it was determined that four instances were coincidental events, whereas three others were considered vaccine-related cases (Table 3).

## 4. Discussion

The PCV13 has been globally embraced and is instrumental in mitigating the risk of pneumonia outbreaks. Since 2017, Hangzhou has provided voluntary vaccination services to children over 1.5 months of age. With increasing awareness of this vaccine over time, its acceptance has shown an annual upward trend. This study was conducted to evaluate the safety of the PCV13, aiming to fully grasp its safety characteristics and provide more reliable vaccination recommendations for the public. A detailed analysis was conducted regarding the adverse events reported by the NAEFISS system. The outcome demonstrated no substantial increase in the risk level for predetermined adverse events. Most of the reported cases exhibited mild symptoms, most of which were self-limiting and relieved within a short time. Common symptoms included fever, injection site reactions (swelling or pain), and gastrointestinal reactions (vomiting or mild diarrhea). These symptoms are usually consistent with common childhood illnesses and conditions in this age group [13]. The occurrence of these mild symptoms did not pose a serious threat to the health of the vaccinated participants, which is consistent with other studies and further confirms the good safety performance of the PCV13 [11,21,22,23,24].

Our study indicated that the AEFI reporting rate for the PCV13 was 27.06 per 10,000 doses. Although this rate aligns closely with previous studies on PCV13 safety post-licensure in China using passive surveillance systems, the reporting rate for severe cases was lower than that reported in the literature [14,25,26]. In the context of temporal trends, there was a low incidence of reported AEFI cases following the administration of the PCV13 in Hangzhou during the first quarter of the year, accompanied by a correspondingly low reporting rate. This phenomenon may be attributed to colder temperatures and thicker clothing during this period, potentially masking mild symptoms such as injection site reactions and leading to delayed detection and reporting by vaccine recipients or their caregivers.

Two types of vaccines with different carrier proteins were included in our study, and the results showed that no statistically significant difference in the reporting rate of AEFIs between the two types, consistent with previous studies [25]. The results of previous research indicated that the reporting rate of AEFIs after PCV13-TT vaccination is not higher than that of the PCV7 or PCV13 with CRM as the carrier. However, owing to the limited use of the PCV13-TT in a few countries and regions such as mainland China, Morocco, and Indonesia, the safety difference between the two types is to be further observed and analyzed.

Wysocki et al. found that the symptoms of local tenderness gradually increase with increasing age [27]. The AEFI reporting rate of children older than 2 years of age was higher than that of other age groups in our study. This phenomenon may be closely related to differences in immune system maturity among different age groups [27,28]. We hypothesize that as children age, a change in immune system responses may affect the occurrence of adverse reactions after vaccination. Additionally, many studies have highlighted significant differences in AEFI reporting rates between different vaccination doses, which is consistent with our findings. According to the literature reports by Zhang et al., as well as our actual research data, the AEFI reporting rate showed a gradual decrease with an increase in basic immunization doses [11,27,29,30]. This phenomenon may be related to parents’ greater attention to adverse reactions after the initial vaccination, which improves the sensitivity of reporting [30]. This increased attention and sensitivity may lead to more adverse reactions being captured and reported. Regarding the time interval dimension, our study found that most AEFI case reports were concentrated within 0–1 days after vaccination. This finding can be explained by the short interval between vaccination and the most common AEFIs, such as fever or injection site reaction, likely caused by local inflammatory reactions or central thermoregulation dysfunction. These adverse reactions usually occur shortly after vaccination, as described in the literature [31,32,33]. Therefore, monitoring and reporting of AEFIs should be strengthened shortly after vaccination, especially within the 0–1-day timeframe, to ensure timely identification and proper treatment of possible adverse reactions.

Fever, a common adverse reaction after vaccination, is not unique to a specific vaccine but is a common phenomenon in the immune process. In the cases included in this study, fever was the most frequently reported symptom and showed an ROR signal, consistent with the conclusions of several studies on adverse reactions to the PCV13 [11,13,24]. Both the PCV13-CRM197 and the PCV13-TT consist of a variety of polysaccharides combined with carrier proteins. These carrier proteins are heterologous to the body, possess biological activity and may trigger a systemic febrile response [32]. Crying is a common response to painful stimuli in children. Vaccinations can also lead to prolonged or abnormal crying in neonates and infants, with differences in incidences potentially associated with various factors, such as different vaccination sites or methods, vaccine types, and needle sizes [33,34,35,36]. The primary distinction between crying included in AEFIs and a child’s typical crying is the prolonged duration (exceeding 3 h) and persistence following vaccination, without any other explanatory factors such as fever or injection site inflammation. This type of crying often triggers significant concern for the vaccine recipient’s caregiver. According to the instructions for the PCV13, the preferred injection site for infants is the anterolateral thigh (lateral femoral muscle), where most of the children in this study were injected. The results of a study comparing the safety of different injection sites showed that the incidence of fever and abnormal crying in the lateral femoral muscle group was higher than that in the upper arm deltoid group [36]. Therefore, the vaccine composition and injection site may be the key causes of these two positive signals. Additionally, since the PCV13 is a self-paid vaccine with a relatively high price, it can be speculated that parents who are willing to pay for the vaccination may pay extra attention to the physical reactions of their children post-vaccination. Local pain at the injection site and abnormal crying caused by general discomfort are easier to detect and report under this extra attention. However, the two positive signals detected in this study did not correspond to rare or severe vaccine-related adverse events. Based on the current results, the PCV13 exhibited favorable safety and tolerability.

Seven severe cases were observed in this study. After considering factors such as onset time, other potential causes of the illness (e.g., viral infections), and medical history, three cases were diagnosed by an expert panel as related to vaccination. The diagnoses included febrile seizure, allergy, and thrombocytopenia. The most probable cause of febrile seizures following PCV13 vaccination is an increase in body temperature after immunization [11]. Although febrile convulsions have been observed in multiple studies, researchers consider that the absolute number of cases is small, and febrile convulsions are usually self-limiting with a good prognosis. Therefore, preventing the occurrence of febrile convulsions should not deter parents or caregivers from vaccinating their children [5,11,12,13]. Allergic reactions are common adverse events associated with immunizations, and pediatricians are not surprised by the occurrence of thrombocytopenia in children, given its known association with vaccination [37].

Our study also documented a case of Kawasaki disease, an exceedingly rare occurrence within the NAEFISS system, irrespective of the administration of the PCV13 or other vaccines. However, this finding warrants careful consideration [18]. The occurrence of vasculitis diseases, including Kawasaki disease, is believed to be associated with the presence of antibodies, circulating immune complexes of antigen-binding antibodies, infections, certain vaccines, and genetic variants [38]. Most studies have shown that although there are reports of AEFI cases of Kawasaki disease following pneumococcal conjugate vaccination, they are considered coincidental events and irrelevant to vaccination [5,39]. However, recent studies have proposed an etiological model of immune complexes binding to Fc receptors, activating immune cells and platelets, and driving Kawasaki disease and multisystem inflammatory syndrome. This model suggests that vaccines are substitute sources of antigens or attenuated pathogens that drive immune complex formation [38]. Therefore, the potential causal association between the PCV13 and Kawasaki disease is worth further study.

This study has certain limitations. The data used in this study were derived from a passive AEFI monitoring database, which has some inherent limitations. These include potential underestimation of adverse events owing to reporting staff concerns, overestimation of adverse events related to the new vaccine focus, and lack of a known baseline incidence rate for adverse events owing to the absence of control groups [18,19]. Passive AEFI monitoring relies solely on case data for risk calculation and serves as an indicator of potential safety signals. However, these findings require verification and confirmation in case-control and cohort studies.

## 5. Conclusions

Based on our research findings between 2020 and 2023, there may be a seasonality in the reports of adverse reactions to the PCV 13, and lower reported numbers and rates are typically seen in the first quarter of each year. Following the administration of the 13-valent pneumococcal vaccine, there were relatively higher incidences of fever and persistent crying in children compared to other vaccines. However, other local symptoms and systemic reactions were well-tolerated. Only seven severe cases were reported, and no unexpected safety issues arose in terms of its safety profile. Overall, the administration of the PCV13 in children demonstrated good safety and tolerability. However, further follow-up and in-depth research are required to assess the long-term safety disparities between the PCV13TT and PCV13-CRM197, as well as severe AEFIs such as Kawasaki disease.

## Figures and Tables

**Table 1:** Features and reporting rate of adverse events following immunization (AEFI) cases related to 13-valent pneumococcal conjugate vaccine (PCV13) from 2020 to 2023 in Hangzhou, China (*N* = 2564).

Variables		No. of AEFI Case	Reporting Rate *	Chi-Squared	*p*-Value
Sex	male	1431	29.11	15.89	<0.05
	female	1133	24.85		
Age (months)	1.5–6	1600	27.83	11.29	<0.05
	7–12	315	29.19		
	13–24	590	24.16		
	>24	59	29.97		
Type of PCV13	CRM197	1629	27.13	0.03	>0.05
	TT	935	26.93		
Injection site	arm	562	22.73	23.21	<0.05
	leg	2002	28.59		
Dose number	1	887	34.11	95.12	<0.05
	2	684	28.59		
	3	474	20.57		
	4	519	23.83		
Type of reporter	Vaccination clinic	2499	26.37	2313.72	<0.05
	CDC	65	0.69		
Cause-specific classification	Vaccine product-related reaction (minor)	2544	26.85	5013.39	<0.05
	Vaccine product-related reaction (severe)	3	0.03		
	Coincidental event	17	0.18		
Severity	Serious	7	0.07	2539.54	<0.05
	Non-serious	2557	26.99		
Onset from vaccination (days)	0–1	2398	25.31	8671.85	<0.05
	2–3	80	0.84		
	4–7	32	0.34		
	8–14	32	0.34		
	>15	22	0.23		
Total		2564	27.06		

*: per 10,000 doses.

**Table 2:** Clinical diagnosis of AEFI cases related to PCV13 from 2020 to 2023 in Hangzhou, China (*N* = 2564).

Clinical Diagnosis	No. of AEFI Cases				Reporting Rate *	ROR-1.96SE
	Non-Serious Cases		Serious Cases			
	Vaccine Product-Related Reaction	Coincidental Event	Vaccine Product-Related Reaction	Coincidental Event		
Fever **	1228	8	0	0	13.04	1.64 ^#^
Injection site reaction	1207	0	0	0	12.74	0.75
Decreased appetite	3	0	0	0	0.03	0.45
Gastrointestinal reactions **	24	1	0	0	0.26	0.33
Persistent crying ***	17	0	0	0	0.18	1.61 ^#^
Feebleness	3	0	0	0	0.03	0.08
Cough	0	3	0	0	0.03	0.19
Angioneurotic edema	2	0	0	0	0.02	0.20
Rash/urticaria	57	1	0	0	0.61	0.24
Allergic reactions (other)	0	0	1	0	0.01	0.02
Cardiovascular	0	0	0	1	0.01	0.03
Hypotonic hyporesponsiveEpisode	1	0	0	0	0.01	0.06
Febrile seizures	0	0	1	1	0.02	0.27
Nervous system reactions (other)	2	0	0	1	0.03	0.22
Thrombopenia	0	0	1	0	0.01	0.03
Kawasaki disease	0	0	0	1	0.01	0.28

*: per 10,000 doses; ^#^: Positive signals. ** Fever: a body temperature reaching or exceeding 37.5 °C (99.5 ℉); Gastrointestinal reactions included vomiting and diarrhea. *** Persistent crying: according to the Brighton Collaboration’s case definition, persistent crying as an AEFI in infants and children should include a. crying continuously and likely to be unaltered for >3 h or b. crying >3 h or likely to be continuous [20].

**Table 3:** Features of serious AEFI cases following PCV13 vaccination from 2020 to 2023 in Hangzhou, China (*N* = 7).

Serious AEFI Cases	Features								
	Report Time	Sex	Age (Months)	Type of PCV13	Dose Number	Injection Site	Onset from Vaccination (Hours)	Clinical Diagnosis	Cause-Specific Classification
Case 1	Jun 2021	Male	36.57	TT	1	Arm	28	Allergic reactions	Vprr *
Case 2	Nov 2021	Male	3.40	CRM197	2	Leg	346	Thrombocytopenia	Vprr *
Case 3	Mar 2022	Male	2.67	TT	1	Leg	4	Kawasaki disease	Coincidental event
Case 4	Jun 2022	Male	5.93	TT	2	Arm	1	Nervous system reactions (epilepsy)	Coincidental event
Case 5	Nov 2022	Female	15.57	CRM197	4	Arm	8	Febrile seizures	Coincidental event
Case 6	Sep 2023	Female	2.87	CRM197	2	Leg	7	Cardiovascular (myocarditis)	Coincidental event
Case 7	Nov 2023	Male	5.87	CRM197	3	Leg	9	Febrile seizures	Vprr *

* Vprr is short for “vaccine product-related reaction”.

## Data Availability

All data are unavailable because of privacy or ethical restrictions.
